# Assessing the robustness and clinical evaluation of a deep−learning segmentation model for head and neck cancer

**DOI:** 10.3389/fonc.2026.1731007

**Published:** 2026-02-13

**Authors:** Daniel H. Schanne, Léandre Cuenot, Sarah Brüningk, Mauricio Reyes, Olgun Elicin

**Affiliations:** 1 Department of Radiation Oncology, Inselspital, Bern University Hospital and University of Bern, Bern, Switzerland; 2 ARTORG Center for Biomedical Engineering Research, University of Bern, Bern, Switzerland

**Keywords:** autosegmentation, deep learning, head and neck cancer, PET/CT, robustness

## Abstract

**Background and purpose:**

Deep learning (DL)-based autosegmentation has improved delineation of organs at risk in radiotherapy for head and neck cancer (HNC). However, automated segmentation of gross tumor volumes (GTVp, GTVn) remains challenging, and robustness under real-world imaging conditions is insufficiently characterized. This study evaluates the robustness and clinical usability of a DL-based PET/CT segmentation model for HNC under clinically relevant perturbations.

**Materials and methods:**

A 3D Dynamic U-Net was trained on the public HECKTOR 2022 dataset (474 training, 50 test cases). Synthetic perturbations (noise, blur, ghosting, bias-field, spike noise, and motion) were applied to PET and CT images at varying severity levels, generating 36 variants per patient. Segmentation quality was measured using Dice score, Hausdorff Distance, and accuracy. Clinical usability was assessed for 50 baseline and 18 perturbed cases by two clinicians using a five-point Likert scale. Radiomic features were correlated with robustness metrics.

**Results:**

Baseline Dice scores were 0.766 (GTVp) and 0.698 (GTVn). Performance dropped significantly under spike noise and bias-field artifacts, especially for GTVn. Clinical usability remained high for GTVp (77.8%) but declined to 27.9% for GTVn under severe perturbations. Lesion volume and surface complexity positively correlated with robustness degradation, while high PET contrast offered protective effects against certain perturbations.

**Conclusion:**

DL-based PET/CT segmentation models for HNC show strong baseline performance and robustness for primary tumors. However, nodal tumor segmentation remains vulnerable to specific image artifacts. Enhancing robustness through targeted data augmentation and validation under variable conditions is essential for clinical integration.

## 1 Introduction

Head and neck cancer (HNC) is the sixth most common malignancy globally, with radiotherapy (RT) constituting one of the primary therapeutic modalities (1). RT planning is complex and requires precise delineation of target volumes and organs at risk (OAR), traditionally a manual and labor-intensive task performed by radiation oncologists (2–4). Recent advancements in deep learning (DL)-based autosegmentation models have significantly impacted this step, achieving reliable OAR segmentation with few manual corrections when no gross changes in anatomy are present (5). However, DL segmentation of target structures remains challenging. Gross tumor volumes (GTV) often present as irregularly shaped lesions that can cross anatomical boundaries and infiltrate adjacent structures, complicating automatic segmentation. Clinical target volumes (CTV), which encompass regions of suspected microscopic tumor spread, rely on extensive domain knowledge of tumor biology and anatomical spread patterns, posing additional hurdles for automated methods. Anatomical changes following pretreatment procedures, such as surgery or chemotherapy, further complicate accurate delineation (6, 7).

Despite these challenges, significant progress has been achieved in DL-based autosegmentation for HNC (8, 9). Recent literature demonstrates promising results, particularly regarding the integration of multimodal imaging like computed tomography (CT) and fluorodeoxyglucose positron emission tomography (FDG-PET). Recently, the HECKTOR (Head and Neck Tumor segmentation and Outcome prediction in PET/CT images) challenges have played a pivotal role in driving this progress by providing standardized, annotated datasets and evaluating model robustness and clinical relevance. For instance, in the HECKTOR 2022 challenge, the winning ensemble achieved Dice similarity coefficients of 0.788 for primary tumors (GTVp) and nodal metastases (GTVn) segmentation, underscoring the capability of advanced DL architectures to approach clinical standards in segmentation accuracy (10).

Despite these successes, critical gaps remain. First, the robustness of DL segmentation models under realistic clinical perturbations, such as anatomical changes, patient movement, imaging noise, or varying acquisition protocols, remains insufficiently characterized. Model performance can decline when faced with data of different quality, from changed imaging equipment, or from new sources, underscoring the need for rigorous robustness evaluations. Recent work suggests that aggressive data augmentation can improve network resilience to imaging variability (11, 12). Furthermore, conventional DL metrics, such as the Dice coefficient or Hausdorff distance (HD), might not fully capture the clinical utility and acceptability of segmentation outputs from the clinician’s perspective (13). For clinical implementation, a DL model must demonstrate not only strong quantitative performance but also high clinical usability and robustness to image quality perturbations commonly encountered in clinical routine.

This study addresses these gaps by rigorously evaluating the performance and robustness of a 3D Dynamic U-Net-based DL segmentation model trained on the publicly available HECKTOR 2022 PET/CT dataset (https://hecktor.grand-challenge.org/Data/). Specifically, we assess the robustness of the segmentation performance under various synthetic perturbations representing clinically relevant image degradation. Additionally, we correlate traditional DL metrics with clinical grading by experienced HNC-specialist radiation oncologists, providing direct insights into the clinical relevance and usability of DL segmentation outputs. By systematically analyzing the correlation between image-derived radiomic features and segmentation robustness, we also aim to identify lesion-specific factors influencing model stability.

In contrast to performance-driven studies that primarily focus on improving algorithms, our goal here is to establish a clear and reproducible characterization of model robustness under realistic perturbations. By defining the boundaries of current state-of-the-art segmentation in head and neck cancer, we provide a factual basis for future methodological work on mitigation strategies, while keeping the present study focused on systematic evaluation and clinical relevance.

## 2 Materials and methods

### 2.1 Dataset and reference contours

We used the public MICCAI HECKTOR 2022 PET/CT dataset (524 patients from nine European/North−American centers: https://hecktor.grand-challenge.org/Data/). Expert-contoured structures of the GTVp and GTVn provided in the dataset served as the reference standard. Because the data are fully anonymized, additional ethics approval was waived.

A stratified 90 / 10 split yielded a development cohort of 474 patients and an internal hold−out cohort of 50 patients. Development images were trained in five−fold cross−validation; the 50 hold−out cases were reserved exclusively for robustness and clinical−grading experiments. These were categorized into two groups: one consisting of baseline images (without perturbations), representing 32 images, and the other comprising perturbed images modified by the three perturbations yielding the largest performance decrease. For each perturbation and modality, three cases were selected, resulting in a total of six cases for each of the three perturbations, accounting for the final 18 cases (i.e, 3 cases x 3 perturbations x 2 modalities).

### 2.2 Pre−processing and augmentation

Aspects of the preprocessing procedure and further data augmentation were adapted from the methods employed by the winning team of the HECKTOR Challenge 2022 (10). In brief, PET volumes were first resampled to the native CT matrix (524 × 524 px) and then both modalities were interpolated to 1 mm³ isotropic voxels. A head−centered crop of 200 × 200 × ≤ 310 voxels removed irrelevant lower−body anatomy.

CT densities were clipped to ±3 SD, min−max scaled to [0, 1]; PET SUVs were z−normalized. Training data underwent random affine jitter, flips, and CT−only density transforms (Gaussian noise, smoothing, contrast, shift). All augmentations were applied with an occurrence probability of 20%. Training patches measured 192 × 192 × 192 voxels and were centered based on labels, with a 10% probability of being centered on background, 45% on primary tumors, and 45% on nodal tumors. In cases where only one tumor type was present, the sampling probability for that tumor was increased to 90%.

### 2.3 Network and training

A 3−D Dynamic U−Net, implemented in MONAI (14), was adopted for the task. The model features six encoder–decoder stages, with imaging information inputted via two channels (CT, PET), and yields three probability maps (background, GTVp, GTVn). Additionally, batch normalization was implemented in conjunction with residual blocks to enhance training stability and model performance. The model was trained using a hybrid loss function that combined Dice and cross-entropy losses. A five-fold cross-validation strategy was used following common practices to enhance model generalization. Training parameters were set based on literature reports of previous winning entries of the Hecktor challenge: AdamW optimizer, learning rate of 1e-4, weight decay of 3e-5, batch size of 2. Training lasted 100 epochs per fold with mixed−precision floating point on a workstation−class NVIDIA A100 GPU. To ensure the final model was robust and representative of state-of-the-art performance, inference was performed using an ensemble of the five models trained during the 5-fold cross-validation. The final segmentation masks were generated by averaging the softmax probability maps from all five folds before applying the 0.5 threshold (obtained through the validation set. This ensembling strategy aligns with the methodology of top-performing teams in the HECKTOR challenge.

### 2.4 Inference

Each test volume was forwarded once (no test−time augmentation). Soft−max probabilities were thresholded at 0.5; small, isolated components were retained, as validation showed < 2% false positives.

### 2.5 Robustness protocol

Six TorchIO (15) perturbations, Gaussian and spike noise, bias−field, motion, blur, ghosting, were applied at three severity levels to CT and PET separately, creating 36 variants per patient plus the baseline. Segmentation quality was measured with Dice, HD95%, sensitivity, specificity, and accuracy. Dice loss was defined as 1 - (2|P ∩Y|/(|P |+ |Y |)), where P is the prediction and Y is the ground truth.

Inspired by Boone et al. (16), we assessed model robustness as ΔDice = Dice_baseline – Dice_perturbed. P−values from paired Wilcoxon tests were Benjamini–Hochberg corrected.

### 2.6 Correlation based on texture analysis

Thirteen shape/density descriptors (volume, surface, boundary length, compactness, centroid distance, CT/PET variability, CT/PET contrast, SUVmax, mean CT number, regions, entropy) were extracted with scikit−image. Pearson coefficients (ρ) between each property and ΔDice were computed for every perturbation.

### 2.7 Clinical grading study

Two radiation oncologists with 12 and 17 years of experience in treating HNC graded segmentation usability on a five−point Likert scale (1 = unusable, 2 = Requires significant modifications, 3 = Requires some modifications, 4 = Requires minor modifications, 5 = fully acceptable). All 32 baseline cases and 18 representative perturbed cases (the three most deleterious artefacts) were reviewed.

### 2.8 Statistical analyses

Inter−observer agreement used weighted Cohen’s κ. To better account for the ordinal nature of the Likert scale, relationships between quantitative metrics and clinical grades were assessed using Spearman’s rank correlation (ρ), consistent with the analysis. For the exploratory radiomics analysis, correlations between image features and robustness metrics were computed using Pearson’s coefficients, with p-values adjusted for multiple comparisons using the Benjamini-Hochberg procedure to control the false discovery rate. Code is available at https://github.com/Leandre354/ECEProject for reproducibility.

## 3 Results

### 3.1 Dataset and the patient cohort

The training and test dataset included 524 patients with oropharyngeal cancer drawn from multi-institutional (n=9) cohorts. Median age was 61 years in training (IQR 54–67) and 60 years in testing (IQR 55–64), and the cohorts were predominantly male (82% in both groups). The high HPV-positivity rate (81-95%) further reflects the contemporary profile of HPV-associated oropharyngeal cancers (**Table 1**).

**Table 1:** Summary of patient demographics and clinical characteristics in the HECKTOR training and testing cohorts.

Variable	Training n (%)	Baseline test n (%)
Median age (IQR)	61 (54-67)	60 (55-64)
Male sex	401 (81.8%)	26 (81.2%)
Stage I-II	258 (54.5%)	20 (62.5%)
Stage III-IV	216 (45.5%)	12 (37.5%)
N0	49 (10.3%)	2 (6.3%)
N1	73 (15.4%)	7 (21.9%)
N2*	202 (42.6%)	14 (43.8%)
N2a	7 (1.5%)	1 (3.1%)
N2b	71 (15.0%)	4 (12.5%)
N2c	41 (8.6%)	2 (6.3%)
N3	31 (6.5%)	2 (6.3%)
HPV positive	260 (81.3%)	19 (95%)
HPV negative	60 (18.8%)	1 (5%)
HPV information missing	154	12
Tobacco use	96 (50.8%)	12 (85.7%)
No tobacco use	93 (49.2%)	2 (14.3%)
Missing tobacco use	285	18

AJCC/UICC 7th edition was used for staging.

Data are presented as counts with percentages or median with interquartile range (IQR). Missing data counts are reported for key clinical variables.

*Incomplete N2 subcategorization in the public dataset.

### 3.2 Baseline segmentation performance

Cross−validation yielded a median Dice of 0.766 ± 0.195 for GTVp and 0.698 ± 0.313 for GTVn (development cohort median [IQR] 0.689 [0.353] and 0.719 [0.337], respectively). On the 50−patient hold−out set, the network reproduced these scores within ±0.01. **Table 2** summarizes lesion−level statistics and segmentation failure counts. Median HD95% on the 50−case hold−out were 9.2 mm (IQR: 14.3) for GTVp and 17.6 mm (IQR: 62.5) for GTVn.

**Table 2:** Baseline lesion−level performance.

Structure	N cases	Mean dice	Median dice	IQR_Dice	False negatives	False positives
GTVp	50	0.678	0.766	0.195	4 (8%)	3 (6%)
GTVn	50	0.614	0.698	0.313	5 (10%)	5 (10%)

GTVp, primary tumor gross total volume; GTVn, metastatic lymph node gross total volume.

Values are lesion−level. “False Negatives” = Dice 0 (no overlap). “False Positives” = reference volume 0; the network produced non−zero voxels.

### 3.3 Effect of synthetic perturbations and drivers of robustness

**Figure 1:**
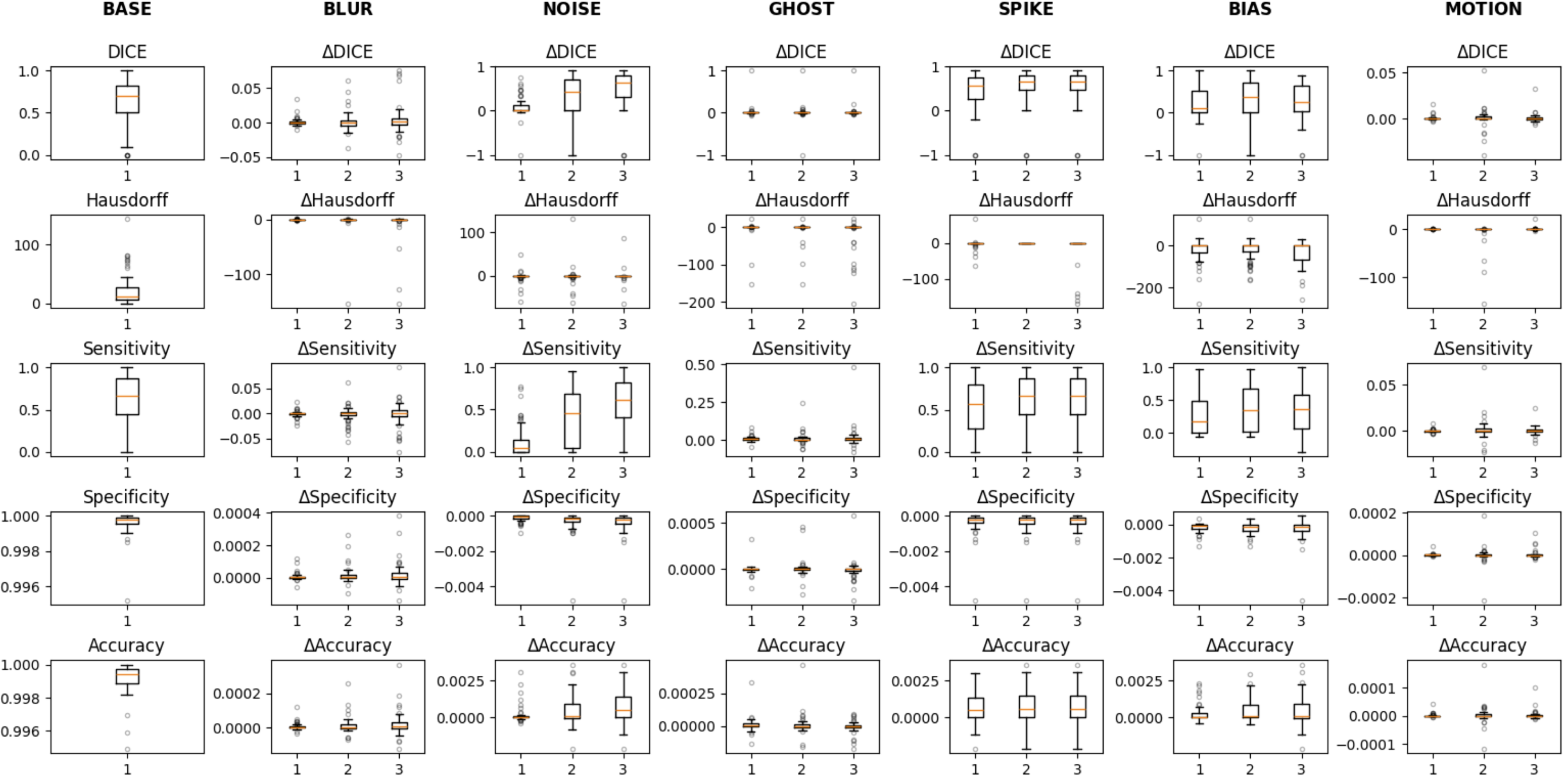
Perturbations applied to nodal tumor contours in the CT. Each of the three box plots corresponds to one of three levels of perturbation severity, providing a visual representation of how segmentation performance is affected at increasing degrees of perturbation.

Across all modalities and structures, blur, ghosting, and rigid motion artifacts produced negligible median Dice (< 0.05, **Supplementary Table 1**). In contrast, spike noise and bias−field shifts were most deleterious, especially for GTVn, occasionally erasing the whole structure. Median ΔDice was markedly higher for GTVn than for GTVp (**Figure 1**, **Supplementary Figures 1**-**3**).

Lesion size drove susceptibility to high−variance artefacts: volume, surface area, and boundary length correlated positively with ΔDice under spike and noise. Compactness and entropy showed weaker effects, while higher PET signal contrast modestly reduced the Dice metric produced by blur and motion artefacts (ρ ≤ 0.39; p = 0.005). Volume, surface area, and boundary length correlated positively with ΔDice under spike and high−variance noise (ρ ≤ 0.62; p < 2e-6). Entropy and compactness showed smaller, yet significant, associations (ρ ≤ 0.39; p = 0.005). On the other hand, PET contrast was mildly protective against blur and motion (ρ ≤ 0.31; p = 0.028), although this did not remain statistically significant after Benjamini-Hochberg correction. No descriptor explained variability under ghosting.

### 3.4 Clinical usability, observer agreement, and metric-grade linkage

**Figure 2:**
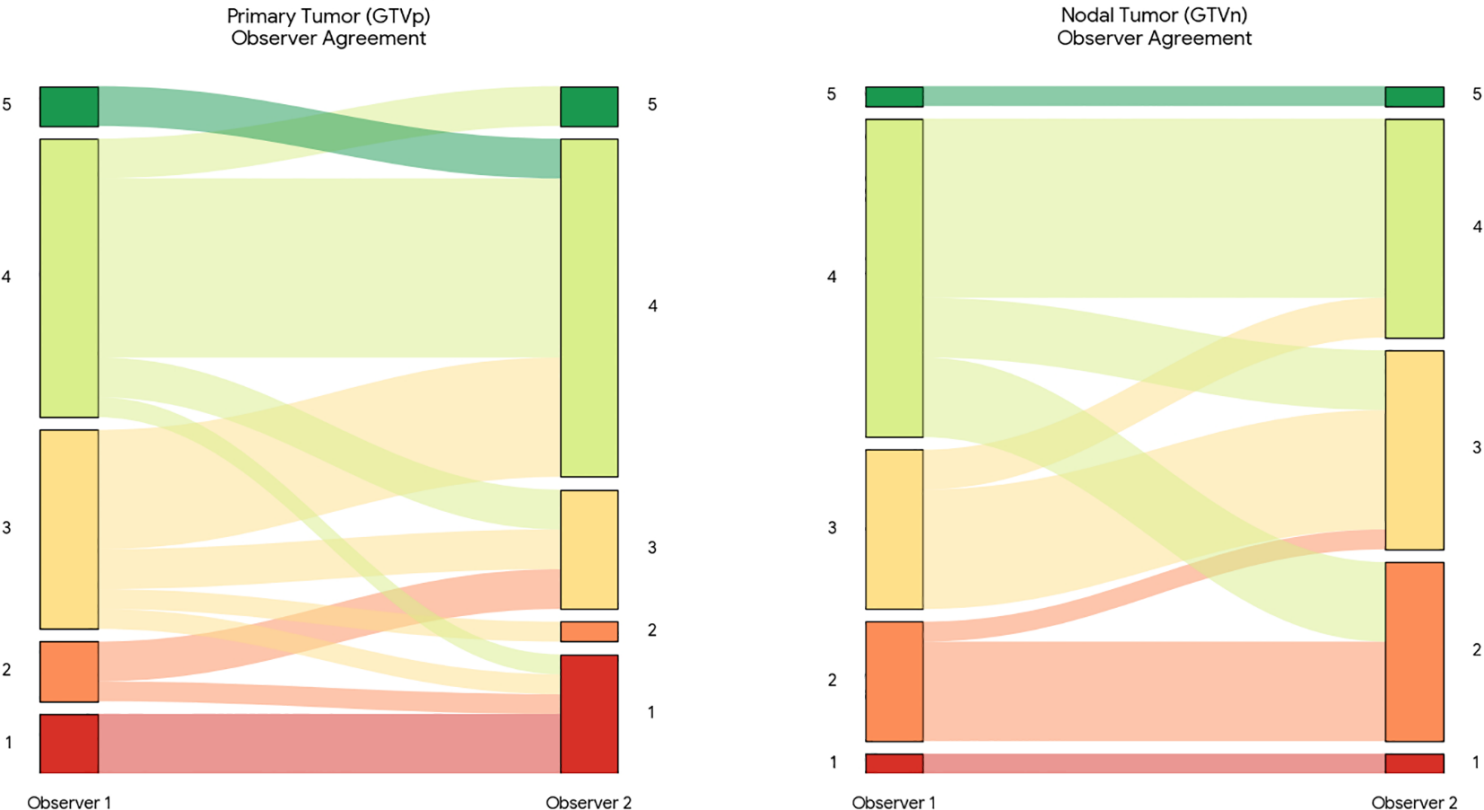
Sankey diagram illustrating inter-observer variability in clinical usability scoring. The flow of cases represents the correspondence between usability scores assigned by Observer 1 (left) and Observer 2 (right) for primary tumors (GTVp) and metastatic lymph nodes (GTVn) on the unperturbed test set. The width of the bands is proportional to the number of patients. Colors correspond to the assigned Likert score (1 = Red/Unusable to 5 = Green/Fully Acceptable). Straight horizontal bands indicate agreement between observers, while crossing bands highlight discordant scoring.

**Figure 3:**
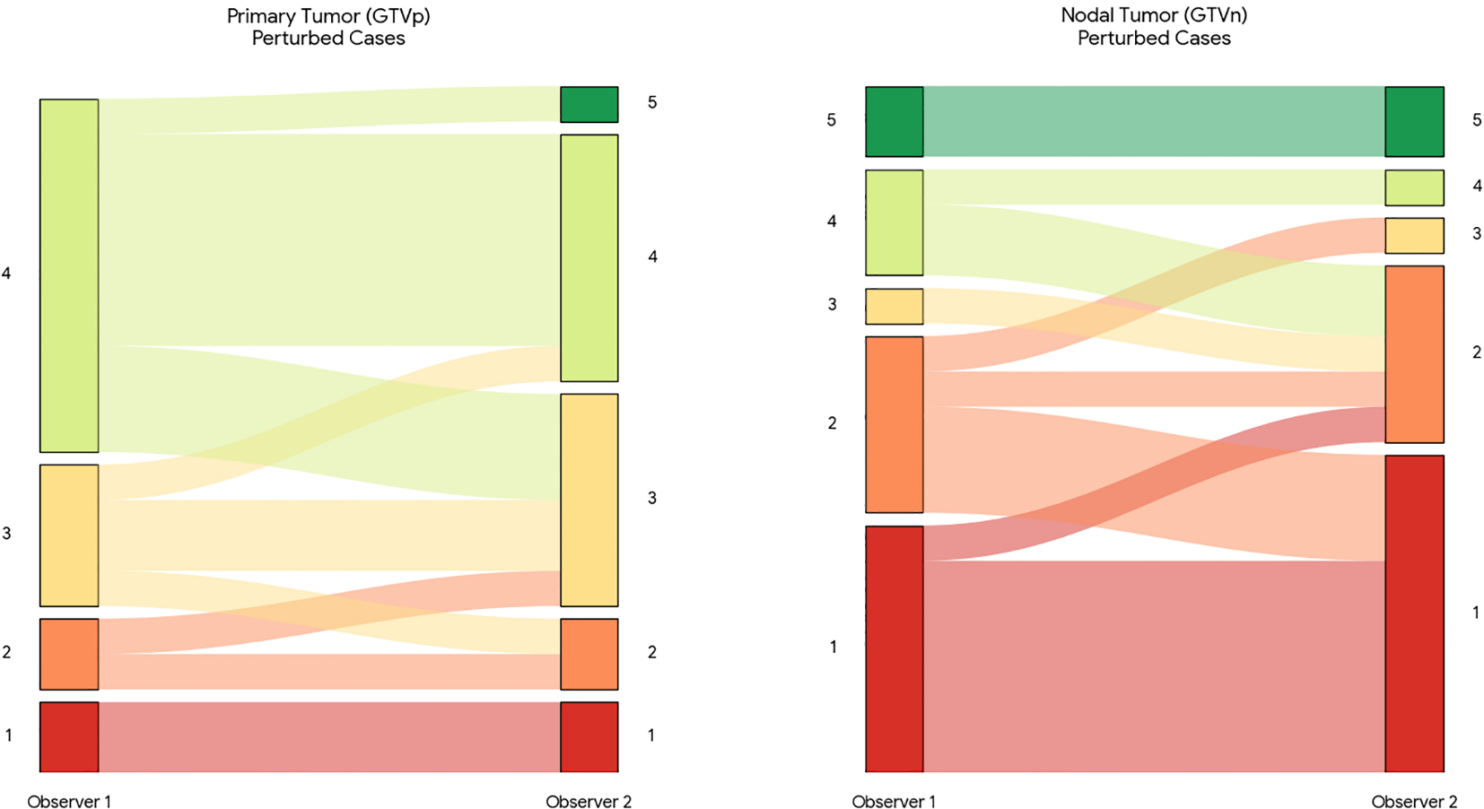
Sankey diagram illustrating inter-observer usability scoring under image perturbations. The diagram displays the flow of clinical usability scores between Observer 1 (left) and Observer 2 (right) for primary tumors (GTVp) and metastatic lymph nodes (GTVn) on the perturbed test subset. This subset includes images degraded by the three most severe artifacts (spike noise, bias field, and motion). Band width is proportional to the number of cases, and colors represent the Likert score (1 = Red/Unusable to 5 = Green/Fully Acceptable). A shift toward lower usability scores (red/orange) is evident compared to the baseline, particularly for nodal volumes, highlighting the sensitivity of these segmentations to image quality degradation.

On unperturbed scans, 79.7% of GTVp and 73.4% of GTVn were rated clinically usable (average of both observers with ≥ 3 points, **Figure 2**). Perturbations reduced GTVn usability to an average of 27.9%, whereas GTVp usability remained high (77.8%), as presented in **Figure 3**.

**Figure 4:**
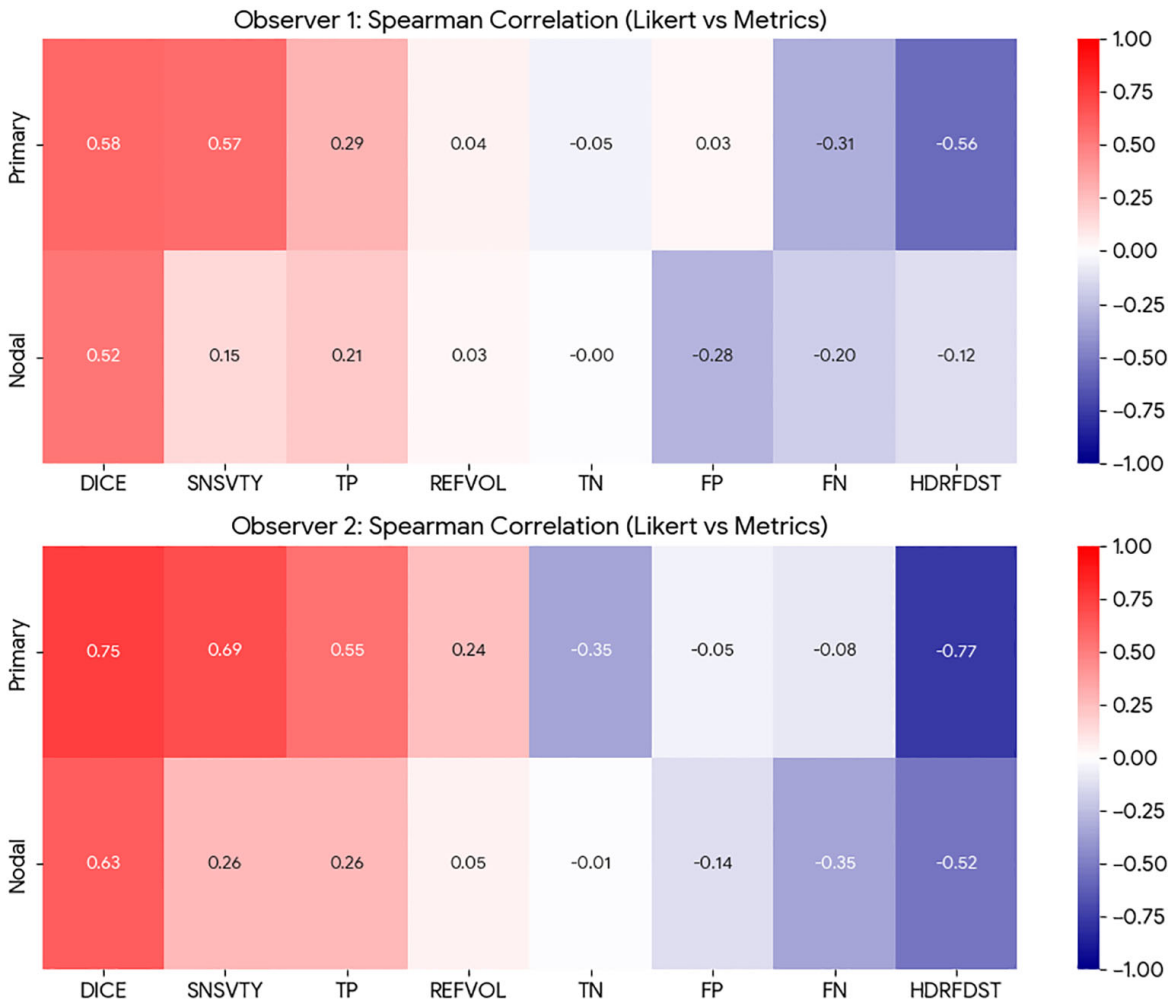
Spearman’s rank analysis between Likert scores and classical segmentation metrics for Observer 1 (Top) and Observer 2 (Bottom) for primary and nodal.

Inter−observer agreement (**Figure 4**) was moderate on baseline images for GTVp (quadratic κ = 0.66) and GTVn (quadratic κ = 0.64), which were increased on the perturbed subset (quadratic κ = 0.83 for both GTVp and GTVn). To further investigate the nature of the disagreements, we analyzed the grading flow between observers (**Figure 2**). This visualization reveals that disagreements were not random; Observer 2 was systematically stricter in evaluating nodal targets (GTVn) compared to Observer 1, frequently assigning lower usability scores to the same contours. This suggests that the reported usability rates for nodal volumes are conservative estimates.

### 3.5 Qualitative failure modes

Spike and bias artefacts either erased the lesion completely or fragmented it into noise-like islands. Missed lesions (Dice=0) occurred in five cases affecting GTVn (10%) and four GTVp (8%), while false positive detections (reference volume = 0 but Dice > 0) occurred in five cases affecting GTVn (10%) and three GTVp (6%). Additionally, severe motion perturbation in PET produced spurious focal uptake that mimicked pathological cervical nodes, likely due to misregistration artifacts that shift normal physiological uptake (e.g. sternocleidomastoid or laryngeal muscles) into the nodal region.

A detailed master’s thesis of the project can be downloaded via https://github.com/Leandre354/ECEProject/blob/main/Doc/MscThesis.pdf, containing additional information, tables and figures.

## 4 Discussion

Our study assessed the robustness and clinical applicability of a deep learning-based autosegmentation model for GTVp and GTVn segmentation in HNC using the publicly available MICCAI HECKTOR 2022 PET/CT dataset. Several key findings emerged from our analyses:

Firstly, the segmentation model demonstrated robust performance, overall, particularly against common imaging perturbations such as blurring, ghosting, and rigid motion artifacts. These perturbations induced negligible losses in segmentation accuracy, as quantified by the median ΔDice values consistently below 0.05. However, the model exhibited marked vulnerability to spike noise and bias-field artifacts, especially in GTVn segmentation. These artifacts frequently caused segmentation failures, either completely erasing lesion predictions or fragmenting them into clinically irrelevant islands. Notably, this disproportionate impact on GTVn is consistent with the general observation that smaller, lower-contrast targets are more challenging to segment reliably (9). These results underscore the importance of further improving model robustness through targeted data augmentation strategies designed to mimic and counteract noise and density distortions. Incorporating stronger and more diverse augmentation has been shown to bolster model performance under such conditions in other imaging contexts (11, 12). Future work should focus on augmenting the training process with simulated spike noise and bias-field corruptions to harden the model against these failure modes.

While our work does not propose new mitigation strategies, its novelty lies in systematically quantifying the vulnerabilities of a clinically relevant segmentation task under controlled and reproducible perturbations. To our knowledge, this is the first robustness study in head and neck cancer autosegmentation that directly correlates algorithmic performance with clinician grading. By precisely documenting which perturbations critically impact performance (e.g., spike noise, bias-field), we establish a factual basis for future methodological improvements. We deliberately chose to focus this study on robust characterization rather than intervention, so that subsequent works can build on these findings with targeted augmentation, uncertainty estimation, or task-tailored architectures. In this sense, our contribution is complementary to performance-oriented studies and provides an essential reference for understanding model limitations in real-world clinical deployment.

Secondly, we identified certain radiomic properties of lesions that influenced segmentation robustness. In our analysis, larger lesions (greater volume and surface area) and those with higher shape complexity (e.g. entropy of density) showed increased susceptibility to perturbations, particularly to spike noise and high-variance noise. This somewhat counter-intuitive finding suggests that while larger tumors are easier to segment under normal conditions, they present a larger canvas on which noise can induce errors (for instance, by creating false fragmentations within an otherwise continuous volume). By contrast, lesions with higher inherent FDG uptake contrast were less affected by blurring and motion, presumably because a strong tumor–background signal helps the model maintain accurate boundaries even when images are slightly degraded. These lesion-specific insights can help guide model refinement. They indicate a need for customized training or augmentation strategies that account for tumor characteristics (size, heterogeneity, contrast) to enhance robustness. For example, additional noise-augmentation could be specifically applied to larger tumors during training, and networks could be conditioned or stratified based on lesion volume so that GTVn (which are smaller and more uniform) are handled by models optimized for those properties. However, any added benefit of such an approach remains speculative.

Thirdly, our clinical grading analysis provides a direct real-world context for these quantitative results. Approximately 80% of GTVp and 73% of GTVn were rated as clinically acceptable (score ≥3 on a 5-point Likert scale) by experienced radiation oncologists on unperturbed images. This high baseline acceptability is in line with other recent studies, in which most auto-segmentations required only minor or no edits​ (17). Perturbations, however, had a pronounced effect on clinical usability for GTVn, the fraction of clinically usable GTVn to ~28% under severe degradation, whereas GTVp remained largely robust (≈78% usable) even with added artifacts. This discrepancy underscores the more challenging nature of GTVn segmentation, likely due to smaller lesion size, lower inherent contrast, and greater anatomical variability in lymph node regions​. It also emphasizes the need for heightened attention and possibly dedicated modeling approaches for nodal structures. Indeed, researchers have found that using task-tailored models (e.g. separate networks focused on lymph node levels) can achieve expert-level delineation for GTVn (17). The average Spearman correlation we observed between traditional segmentation metrics (Dice) and the experts’ usability scores (0.67 for GTVp and 0.5 for GTVn) further validates the utility of these metrics as proxies for quality. That said, quantitative metrics alone cannot fully replace clinical judgment and there are cases in our cohort where an adequate Dice score corresponds to a clinically unacceptable contour placement (for example due to violation of a critical structure boundary). Recent work comparing automated metrics to human perception confirms that conventional overlap measures correlate only moderately with expert assessments of contour quality (13). Moreover, recent work (18) has also shown a low correlation between geometry-based metrics, such as Dice, and dosimetry. This highlights the importance of incorporating expert review in the loop and potentially developing new metrics that better capture clinically relevant errors (5), and dosimetric implications.

In comparison to the existing literature, our results reinforce and extend findings from recent HECKTOR challenges and other multi-institutional studies. Our achieved Dice coefficients on the hold-out test set (approximately 0.77 for primary tumors and 0.70 for nodal tumors) align closely with previously reported segmentation performance by state-of-the-art models on similar PET/CT tasks. For example, the top-performing algorithms in the HECKTOR 2022 challenge obtained an average DSC of ~0.80 for GTVp and ~0.78 for GTVn​ (10), and a recent multi-center study reported DSC in the range 0.71–0.78 for primary GTV delineation (9). This concordance suggests that modern DL architectures, such as the 3D Dynamic U-Net used here, are an important step towards the level of accuracy needed for clinical adoption​ (19). Notably, our use of a two-channel 3D U-Net is conceptually in line with the nnU-Net framework, which has demonstrated robust generalization across numerous segmentation benchmarks by automatically configuring U-Net models to a given task (19). However, our study goes beyond prior works by explicitly quantifying robustness under controlled perturbations and by directly correlating algorithm performance with clinician ratings. These analyses provide novel insights that typical challenge reports (which often focus only on clean-scan Dice scores) do not capture, namely, how and why a model might fail in real-world settings and how well its output would be received by end-users. By clarifying these points, we highlight the remaining challenges that must be addressed for reliable deployment of autosegmentation in routine RT planning (e.g. handling image noise and variability, and ensuring outputs meet clinical quality standards).

Several limitations of our work should be acknowledged. First, the absence of MRI data is a notable shortcoming, given that MRI is superior for delineating primary tumor extent in many HNC cases (especially for soft-tissue and perineural infiltration) (20–22). Our PET/CT-only model may thus miss subtleties that an MRI-enhanced model could capture. Future studies should prioritize multimodal imaging integration, particularly incorporating MRI, to further improve segmentation completeness and accuracy for structures where MRI offers additional contrast. Second, our robustness evaluation was performed entirely within the HECKTOR 2022 multi-institutional dataset, using an internal hold-out set rather than a fully independent external cohort. Hence, an external validation remains an important next step to confirm robustness across unseen acquisition protocols. Third, the inconsistent availability of certain patient- and HNC-specific parameters (especially HPV status and smoking history) in the public dataset prevented us from performing subgroup analyses. Such analyses could be informative (e.g. HPV-positive oropharyngeal tumors might be easier or harder to segment due to different morphology and texture), and their absence may limit the generalizability of our conclusions across different patient populations. Fourth, because our development and validation were conducted on a single multicenter public challenge dataset, it remains to be confirmed that the performance and robustness observed will transfer to another, independent external data source. Prior studies have shown that even top-performing models can experience performance degradation when applied to new hospitals or scanner settings (23). To ensure true generalizability, our model would therefore have to be evaluated on external datasets (for example, from other institutions or prospective trials), and potentially fine-tuned, to verify that its accuracy and robustness hold beyond the HECKTOR cohort​. Fifth, the clinical evaluation of perturbed cases was performed on a subset of images selected to represent the most severe artifacts (“stress testing”). This selection introduces a bias towards lower performance and does not necessarily reflect the distribution of image quality found in routine clinical practice. Therefore, the reported usability rates under perturbation should be interpreted as a lower bound of the model’s resilience in worst-case scenarios.

Another limitation concerns the clinical usability study, which relied on the evaluations of two experienced radiation oncologists from the same institution. While this provides valuable expert input, inter-observer variability in HNC contouring is known to be substantial, and including a larger panel of raters could have captured a broader range of clinical practice. Nevertheless, both observers had >10 years of clinical experience in HNC radiotherapy, providing a reliable reference for assessing usability in this initial study.

Furthermore, our analysis was restricted to gross tumor volumes (GTVp and GTVn). Several studies and commercially available software packages have already demonstrated high clinical adoption of OAR autosegmentation in head and neck RT. Future work will therefore extend our robustness analysis to combined target and OAR segmentation within the same pipeline, which would provide a more comprehensive assessment of clinical utility. Finally, we emphasize that geometric metrics like Dice do not always predict clinical or dosimetric significance. As we recently demonstrated in brain tumor segmentation, evaluators often struggle to estimate the dosimetric impact of contouring variations based on geometry alone (24). For example, a complete nodal erasure represents a major dosimetric miss, whereas minor surface irregularities may have negligible therapeutic consequences. Therefore, future validation should extend beyond geometric robustness to assess downstream dosimetric effects and integrate automated quality assurance to streamline clinical workflows (25).

In conclusion, our study confirms the promising clinical utility of DL-based autosegmentation models for HNC while highlighting existing robustness challenges and areas for further improvement. Future research directions should emphasize multimodal imaging integration (e.g. adding MRI for primary tumor delineation), tailored augmentation strategies to increase robustness, and comprehensive clinical validation on independent external cohorts to confirm generalizability. Availability of accurate and complete clinical data will be a prerequisite to achieve this goal. Additionally, combining accurate segmentation with downstream classification models for high-risk features could enhance clinical decision-making. For instance, coupling such a segmentation approach with a convolutional neural networks classifier for extranodal extension may allow automated detection of nodal extracapsular spread, an application where recent studies have shown promising results on CT imaging (26, 27). By addressing these next steps, we move closer to reliable and clinically applicable automated segmentation tools that can streamline RT planning and improve patient care.

## Data Availability

Publicly available datasets were analyzed in this study. This data can be found here: https://hecktor.grand-challenge.org/Data/. The code is available here: https://github.com/Leandre354/ECEProject.
