# Supplementary material for: Assessing the robustness and clinical evaluation of a deep−learning segmentation model for head and neck cancer
Source: Front Oncol. 2026 Feb 13;16:1731007. doi: 10.3389/fonc.2026.1731007 (PMC12945774; doi:10.3389/fonc.2026.1731007)
Supplement: Supplementary file 1 [file DataSheet1.docx]

Supplementary Material

# Supplementary Table

**Supplementary Table:** Median ΔDice per artefact

| **Artefact** | **Median_ ΔDice** | **IQR** |
| --- | --- | --- |
| Bias CT | 0.281 | 0.653 |
| Bias PET | 0.098 | 0.502 |
| Blur CT | 0.001 | 0.008 |
| Blur PET | 0.004 | 0.042 |
| Ghost CT | 0.001 | 0.012 |
| Ghost PET | 0.003 | 0.023 |
| Motion CT | 0.000 | 0.002 |
| Motion PET | 0.000 | 0.007 |
| Noise CT | 0.235 | 0.659 |
| Noise PET | 0.090 | 0.600 |
| Spike CT | 0.668 | 0.398 |
| Spike_PET | 0.598 | 0.768 |

Lists Bias, Blur, Ghost, Motion, Noise, Spike for CT and PET with their median ΔDice and IQR. Values are rounded to three decimal places.

## Supplementary Figures

**Supplementary Figure 1:** Perturbations applied to primary tumor contours in the CT


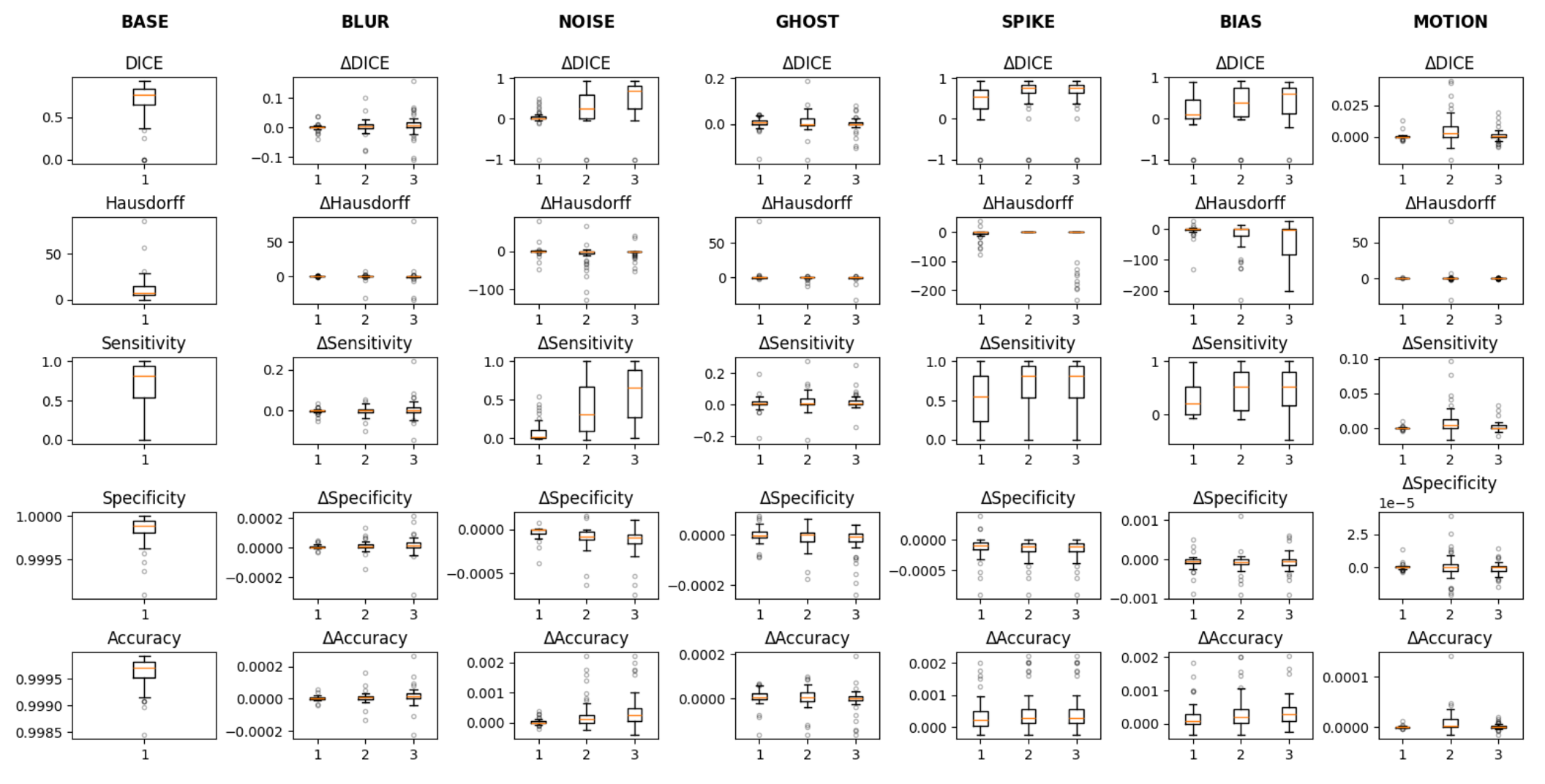


Each of the three box plots corresponds to one of three levels of perturbation severity, providing a visual representation of how segmentation performance is affected at increasing degrees of perturbation.

**Supplementary Figure 2:** Perturbations applied to nodal tumor contours in the PET


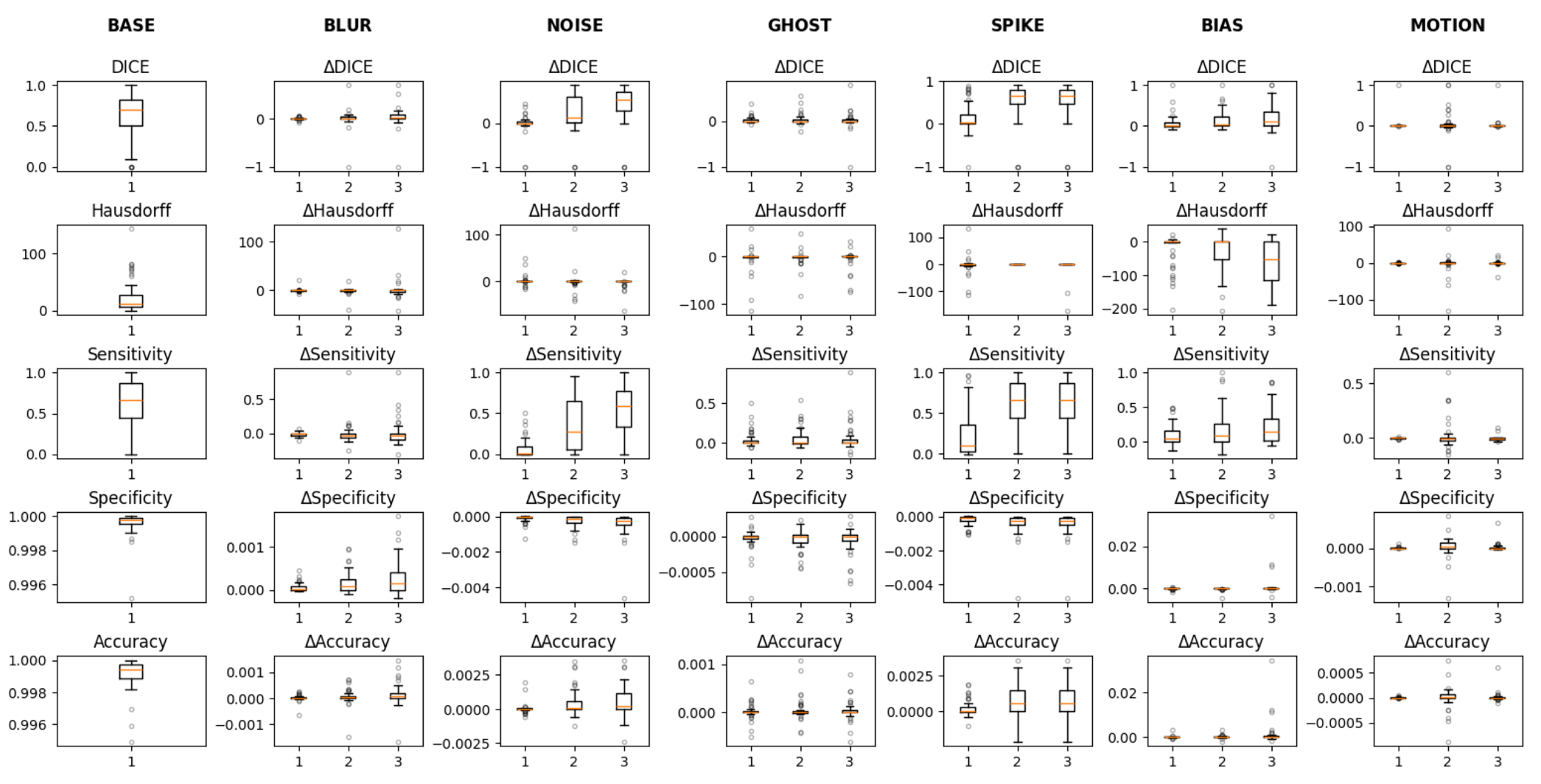


Each of the three box plots corresponds to one of three levels of perturbation severity, providing a visual representation of how segmentation performance is affected at increasing degrees of perturbation.

**Supplementary Figure 3:** Perturbations applied to primary tumor contours in the PET


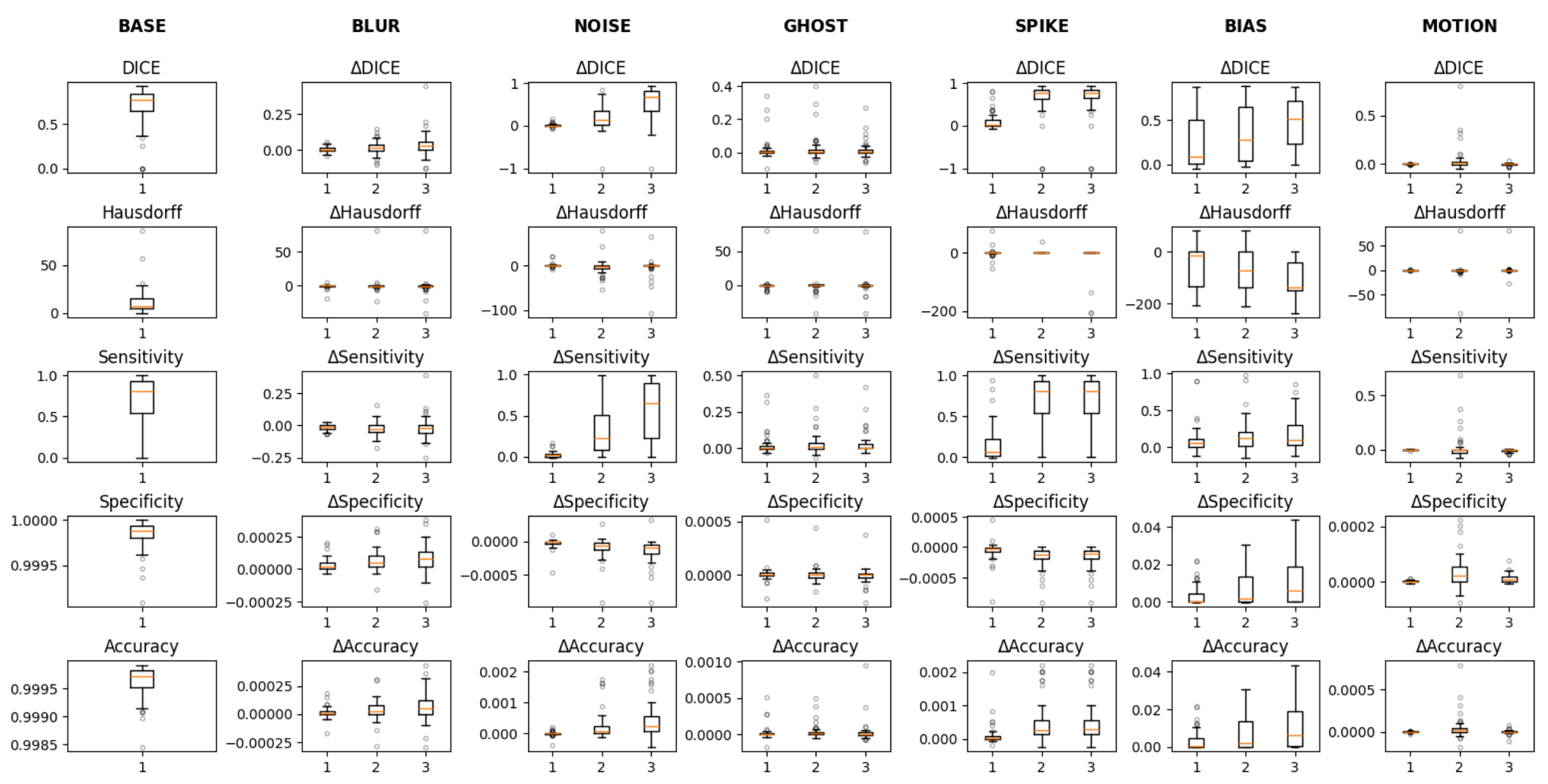


Each of the three box plots corresponds to one of three levels of perturbation severity, providing a visual representation of how segmentation performance is affected at increasing degrees of perturbation.
